# Dual targeting of joint and lung disease: efficacy of tofacitinib plus iguratimod combination in progressive fibrosing rheumatoid arthritis-associated interstitial lung disease

**DOI:** 10.3389/fimmu.2025.1624125

**Published:** 2026-01-16

**Authors:** Yuan Zhu, Yingzi Ye, Wanlin Zhou, Xu Zheng, Weilin Xie, Zhiying Wu

**Affiliations:** 1 Military Treatment Center, the 970th Hospital of the People’s Liberation Army Joint Logistics Support Force, Yantai, China; 2 Health Management Department, the Armed Police Yantai Special Service Sanatorium Center, Yantai, China

**Keywords:** rheumatoid arthritis, progressive fibrosing interstitial lung disease, tofacitinib plus iguratimod, treatment, efficacy

## Abstract

**Objective:**

To evaluate the efficacy and safety of tofacitinib (TOF) plus iguratimod (IGU) in treating progressive fibrosing rheumatoid arthritis-associated interstitial lung disease (PF-RA ILD).

**Methods:**

This historical-controlled study enrolled 28 PF-RA ILD patients (13 received TOF plus IGU; 15 received the biologic/conventional synthetic disease-modifying anti-rheumatic drugs (b/csDMARDs). Disease activity, pulmonary function (PFTs), high-resolution computed tomography (HRCT) scores, and safety were assessed longitudinally and between groups.

**Results:**

Baseline characteristics were comparable (*P*>0.05). The TOF plus IGU group showed significant improvements: C-reactive protein (CRP) decreased (30.5 ± 23.1 to 5.1 ± 3.3 mg/L, *P* < 0.05), erythrocyte sedimentation rate: 46.2 ± 18.8 to 20.1 ± 18.9 mm/h, *P* = 0.012). The disease activity score 28-joint count with CRP declined from high to low activity, and rheumatoid factor titers dropped (79.7 ± 64.2 to 23.4 ± 21.7 IU/mL at 12 months, *P* = 0.023). Similarly, anti-cyclic citrullinated peptide levels declined from 157 ± 57.5 RU/mL to 109.8 ± 32.6 RU/mL at 6 months (*P* = 0.028). Pulmonary function improved, with forced vital capacity increasing from 79.5 ± 12.9% to 85.3 ± 13.6% at 6 months (*P* = 0.008). HRCT fibrosis scores decreased from 9.6 ± 2.5 to 5.1 ± 1.6 (*P* = 0.026). Compared to controls, TOF plus IGU demonstrated superior outcomes: lower CRP (8.5 vs 20.2 mg/L, *P* = 0.002), higher diffusing capacity for carbon monoxide at 3 months (73.1 ± 19.6% vs 61.1 ± 14.5%, *P* = 0.045), and lower fibrosis scores at 12 months (5.1 vs 7.5, *P* = 0.004). At 12 months, imaging stability/regression occurred in 92.3% vs 60.0% (*P* = 0.047). All TOF plus IGU patients tapered prednisone. No thromboembolic events or severe infections occurred.

**Conclusion:**

TOF plus IGU demonstrated dual efficacy in controlling synovitis and lung fibrosis, with a favorable safety profile.

## Introduction

Interstitial lung disease (ILD), a serious extra-articular manifestation of rheumatoid arthritis (RA), is associated with significant mortality and represents a critical challenge in RA management. Epidemiological studies report a highly variable prevalence of RA-ILD, ranging from 3% to 67%, with meta-analyses indicating that RA-ILD increases mortality risk by 2~10 fold compared to RA patients without pulmonary involvement (1–3). As the second leading cause of death in RA patients after cardiovascular diseases, RA-ILD demonstrates substantial clinical heterogeneity, manifesting as distinct high-resolution computed tomography (HRCT) patterns. Usual interstitial pneumonia (UIP), observed in 40~60% of cases, is the most common and prognostically unfavorable subtype, followed by nonspecific interstitial pneumonia (NSIP) (~40%) and unclassifiable patterns (~6%) (4). A meta-analysis of 1,256 RA-ILD patients across 10 cohorts revealed that the UIP pattern confers a 1.6-fold higher mortality risk compared to other subtypes (5).

Approximately 30~40% of RA-ILD patients develop a progressive fibrotic phenotype characterized by worsening lung fibrosis on HRCT, declining pulmonary function, refractory symptoms, and early mortality despite conventional immunosuppressive therapies (6). Therapeutic decision-making remains complex due to the dual challenges of drug-induced pulmonary toxicity from conventional synthetic disease-modifying antirheumatic drugs (csDMARDs) and limited evidence for therapies that effectively halt fibrotic progression (7). This underscores the urgent need for novel treatment strategies targeting fibrotic pathways.

Emerging insights into the pathogenesis of pulmonary fibrosis highlight the critical role of the Janus kinase/signal transducer and activator of transcription (JAK-STAT) pathway (8, 9). Tofacitinib (TOF), an oral JAK1/JAK3 inhibitor with partial JAK2 inhibition, has demonstrated potential in modulating fibrotic processes through its downstream effects on profibrotic cytokines and macrophage polarization. Recent *in vitro* and *in vivo* studies have further elucidated that JAK inhibitors, including tofacitinib, exert combined anti-fibrotic and anti-inflammatory effects by modulating macrophage polarization, which is central to fibrotic progression (10). Preclinical and clinical studies suggest TOF may stabilize lung function and radiographic progression in connective tissue disease-associated ILD, particularly in progressive fibrosing RA-ILD (11–14). In alignment with these findings, a recent multicenter prospective study from France (MAJIK-SFR registry) evaluated 42 RA-ILD patients treated with JAK inhibitors including tofacitinib and demonstrated stability in forced vital capacity (FVC%) and diffusing capacity for carbon monoxide (DLCOSB%) over a median follow-up of 21 months, with HRCT lesions remaining stable in 69% of patients (15). Real-world data from a multicenter observational study also support the stabilization of pulmonary function in RA-ILD patients treated with tofacitinib, with an acceptable safety profile (16). Concurrently, iguratimod (IGU), a novel antirheumatic agent with dual anti-inflammatory and antifibrotic properties, has shown promise in suppressing fibroblast activation and collagen deposition through inhibition of nuclear factor-κB (NF-κB) and transforming growth factor-β (TGF-β) signaling (17–19). A prior clinical study demonstrated that adding iguratimod to glucocorticoid/cyclophosphamide therapy significantly improved lung function and reduced systemic inflammation in RA-ILD patients compared to immunosuppressants alone (20). Recent phase IV trials further support its favorable safety profile in RA patients with pulmonary comorbidities.

While both agents are widely used in China for RA management, no clinical studies have evaluated the combined efficacy of TOF and IGU in progressive fibrosing (PF)-RA ILD. This knowledge gap is particularly significant given the synergistic potential of JAK inhibition and immunomodulation in addressing both inflammatory and fibrotic disease components. To address this unmet need, we conducted an observational study analyzing clinical outcomes in 28 patients with PF-RA ILD treated with TOF and IGU combination therapy, assessing its safety and effectiveness in real-world practice.

## Methods

### Study design and participants

This historical comparator study was conducted at the 970th Hospital of the People’s Liberation Army Joint Logistics Support Force (Yantai, China) from January 2016 to May 2025. Patients diagnosed with PF-RA ILD were prospectively enrolled into the experimental group if deemed suitable for JAK inhibitor therapy. Patients with prior exposure to JAK inhibitors were excluded from both groups. A retrospective control group was selected from PF-RA ILD patients treated with biologic or conventional synthetic disease-modifying anti-rheumatic drugs (b/csDMARDs) during the same period.

All participants fulfilled the following criteria: RA diagnosis: 2010 American College of Rheumatology (ACR)/European League Against Rheumatism (EULAR) classification criteria (21). All enrolled patients had active RA at baseline (Disease Activity Score 28-joint count with CRP (DAS28-CRP) > 2.6). ILD diagnosis: 2002 American Thoracic Society/European Respiratory Society consensus criteria. CT patterns were classified according to the guidelines and relevant literature into UIP, NSIP, organizing pneumonia (OP), or unclassifiable patterns (22). Furthermore, HRCT images for all patients were independently evaluated by two thoracic radiologists blinded to the patient groups.

Progressive fibrosing phenotype: Defined as ≥1 of the following within 24 months prior to enrollment, despite standard care: Relative decline in forced vital capacity (FVC) ≥10%; Relative FVC decline ≥5% accompanied by worsening respiratory symptoms or increased fibrosis on HRCT; Symptomatic deterioration with radiographic progression (6). Exclusion criteria included ILD progression secondary to infections, heart failure, environmental exposures, or overlapping connective tissue diseases.

### Ethical considerations

The study protocol was approved by the Ethics Committee of the 970th Hospital, with the informed consent granted for data collection. All procedures adhered to the Declaration of Helsinki.

### Treatment regimens

Experimental group: Oral TOF (5 mg twice daily) combined with IGU (25 mg twice daily). Concomitant prednisone (≤15 mg/day) was permitted but not mandated. Prior csDMARDs (e.g., methotrexate) were maintained, reduced, or discontinued at the physician’s discretion; no new immunosuppressants were introduced during the study. Control group: Received b/csDMARDs (e.g., cyclophosphamide, tacrolimus, tumor necrosis factor inhibitors) with or without prednisone (≤15 mg/day).

### Outcome measures

Patients underwent standardized assessments at baseline and at 3, 6, and 12 months: RA disease activity: DAS28-CRP: Categorized as high (HDA: >5.1), moderate (MDA: 3.2~5.1), low (LDA: 2.6~3.2), or remission (≤2.6). Inflammatory markers: CRP (mg/L) and erythrocyte sedimentation rate (ESR, mm/h).

Pulmonary function tests (PFTs): FVC% and DLCO SB%, corrected for hemoglobin.

HRCT scores: Semi-quantitative scoring: Three axial slices (aortic arch, tracheal bifurcation, 1 cm above diaphragm) were analyzed for each lung lobe. Ground-glass opacity (GGO) scores for GGO involving the lobe: 0, none; 1, ≤5%; 2, 5% to <25%; 3, 25% to 49%; 4, 50% to 75%; and 5, >75%. Fibrosis scores for reticulation (including intralobular lines), traction bronchiectasis, bronchial dilation, architectural distortion and honeycombing involving the lobe: 0, none; 1, interlobular septal thickening without discrete honeycombing; 2, <25%; 3, 25~49%; 4, 50~75%; and 5, >75% (23, 24). The total GGO score and total fibrosis score were calculated as the sum of the respective scores from all lobes (0~24). Radiographic progression: Defined as ≥10% increase (deterioration), ≤10% change (stability), or ≥10% decrease (regression) in total fibrosis extent (23, 25).

Safety monitoring: Adverse events (e.g., infections, thrombosis) were recorded.

### Statistical analysis

Continuous variables are expressed as mean ± standard deviation (SD) or median (range), depending on data distribution assessed via Shapiro-Wilk test. Between-group differences were analyzed using: Independent t-test (parametric data) or Mann-Whitney U test (non-parametric data). Chi-square test or Fisher’s exact test for categorical variables. Longitudinal changes: Paired t-test or Wilcoxon signed-rank test for within-group comparisons. Statistical significance was set at P < 0.05 (two-tailed). Analyses were performed using SPSS v23.0 (IBM, USA).

## Result

### Baseline demographic and clinical characteristics of PF-RA ILD patients before treatment

Among 142 patients with RA-ILD screened at our center, 31 met the criteria for PF-ILD. After excluding three patients due to concurrent infections, heart failure, or incomplete clinical data, 28 were enrolled, with 13 assigned to the TOF plus IGU group and 15 to the b/csDMARDs control group. Baseline demographic and clinical characteristics were balanced between groups (*P* > 0.05 for all parameters, **Table 1**). The TOF plus IGU group comprised 61.5% males versus 53.3% in controls (*P* = 0.450), with comparable mean ages (70.2 ± 11.4 vs. 68.3 ± 9.6 years, *P* = 0.488). No significant differences were observed in smoking status (46.2% vs. 46.7%, *P* = 0.605), RA duration (4.1 ± 3.1 vs. 4.3 ± 3.6 years, *P* = 0.818), or ILD duration (18.1 ± 12.9 vs. 19.8 ± 10.1 months, *P* = 0.572). The UIP and NSIP was the main CT patterns and the distribution of these was comparable between the two groups (UIP pattern: 61.5% vs. 60.0%; NSIP pattern: 30.8% vs. 33.3%; *P =* 0.625). Comorbidities were prevalent in both groups (92.3% vs. 86.7%, *P* = 0.448). Baseline steroid use (69.2% vs. 73.3%, *P* = 0.546) and methotrexate (46.2% vs. 46.7%, *P* = 0.605), leflunomide (7.7% vs. 20.0%, *P* = 0.248), hydroxychloroquine (23.1% vs. 13.3%, *P* = 0.275), tumor necrosis factor inhibitors (TNFi) (7.7% vs. 13.3%, *P* = 0.448) exposure were similar. Importantly, the baseline HRCT total fibrosis scores were comparable between the two groups (9.6 ± 2.5 vs. 10.9 ± 3.7, *P* = 0.282).

**Table 1:** Baseline demographic and clinical characteristics of patients with PF RA-ILD (n=28).

Variables	TOF+IGU n=13	b/csDMARDs n=15	P-value
Demographics			
Male sex, n (%)	8 (61.5)	8 (53.3)	0.450
Age, years, mean ± SD	70.2 ± 11.4	68.3 ± 9.6	0.488
Smoking history, yes, n (%)	6 (46.2)	7 (46.7)	0.605
Disease history			
RA duration, years, mean ± SD	4.1 ± 3.1	4.3 ± 3.6	0.818
ILD duration, months, mean ± SD	18.1 ± 12.9	19.8 ± 10.1	0.572
HRCT pattern at enrollment, n (%)			
UIP	8 (61.5)	9 (60)	0.625
NSIP	4 (30.8)	5 (33)	
Unclassifiable	1 (7.7)	1 (7)	
Comorbidities, n (%)	12 (92.3)	13 (86.7)	0.448
Treatments at baseline, n (%)			
Steroids	9 (69.2);	11 (73.3)	0.546
Methotrexate	6 (46.2)	7 (46.7)	0.605
Leflunomide.	1 (7.7)	3 (20.0)	0.248
Hydroxychloroquine	3 (23.1)	2 (13.3)	0.275
TNFi	1 (7.7)	2 (13.3)	0.448
Tripterygium glycosides	6 (46.2)	8 (53.3)	0.480

Data are presented as mean ± standard deviation or n (%).

PF, progressive fibrosis; RA, rheumatoid arthritis; ILD, interstitial lung disease; TOF, tofacitinib; IGU, iguratimod; csDMARDs, conventional synthetic disease-modifying anti-rheumatic drugs; HRCT, high-resolution computed tomography; UIP, usual interstitial pneumonia; NSIP, nonspecific interstitial pneumonia; TNFi, tumor necrosis factor inhibitors.

### The RA activity, PFTs, and HRCT scores after treatment

There were no significant differences in follow-up duration (18.8 ± 4.3 vs. 16.7 ± 6.2 months, *P* = 0.133). At follow-up, steroid utilization remained comparable between groups (76.9% vs. 80.0%, *P* = 0.564). In the TOF plus IGU group, glucocorticoid tapering was protocolized with a monthly reduction of 2.5 mg prednisone equivalent where clinically feasible; six of patients on baseline steroids (6/13, 46.2%) successfully discontinued steroid therapy by month 12. The control group received additional therapies including cyclophosphamide (40.0%), tacrolimus (26.7%), and tumor necrosis factor inhibitors (53.3%) (**Table 2**).

**Table 2:** Follow-up duration and treatments (n=28).

Variables	TOF+IGU n=13	b/csDMARDs n=15	P-value
Follow-up duration, months, mean ± SD	18.8 ± 4.3	16.7 ± 6.2	0.133
Treatments at follow-up, n (%)			
Steroids	10 (76.9)	12 (80.0)	0.564
Cyclophosphamide	–	6 (40.0)	–
Tacrolimus	–	4 (26.7)	–
Hydroxychloroquine	–	6 (40.0)	–
TNFi	–	8 (53.3)	–
Tripterygium glycosides	–	9 (60.0)	–

Data are presented as mean ± standard deviation or n (%).

TOF, tofacitinib; IGU, iguratimod; csDMARDs, conventional synthetic disease-modifying anti-rheumatic drugs; TNFi, tumor necrosis factor inhibitors.

In the TOF plus IGU group, inflammatory markers demonstrated progressive reductions: CRP decreased from 30.5 ± 23.1 mg/L at baseline to 8.5 ± 6.6 mg/L at 3 months (*P* = 0.024), 8.9 ± 10.9 mg/L at 6 months (*P* = 0.045), and 5.1 ± 3.3 mg/L at 12 months (*P* = 0.033). ESR declined from 46.2 ± 18.8 mm/h to 20.1 ± 18.9 mm/h (*P* = 0.012), paralleled by significant improvements in disease activity. Rheumatoid factor (RF) titers decreased from 79.7 ± 64.2 IU/mL to 23.4 ± 21.7 IU/mL by 12 months (*P* = 0.023). Similarly, anti-cyclic citrullinated peptide (anti-CCP) levels declined from 157 ± 57.5 RU/mL to 109.8 ± 32.6 RU/mL, with a significant reduction observed at 6 months (*P* = 0.028). Over the 12-month treatment period, high disease activity (HDA) decreased from 53.8% to 7.7%, while CR increased from 0% to 53.8% (*P* < 0.001). Pulmonary function tests revealed sustained improvements in FVC%, rising from 79.5 ± 12.9% at baseline to 85.3 ± 13.6% at 6 months (*P* = 0.008) and stabilizing at 84.4 ± 11.5% by 12 months (*P* = 0.009). DLCO SB% increased from 62.3 ± 11.6% to 70.5 ± 12.1% (*P* = 0.040). HRCT scores indicated substantial fibrosis suppression, with total fibrosis scores decreasing from 9.6 ± 2.5 to 5.1 ± 1.6 (*P* = 0.026) and GGO scores declining from 4.9 ± 2.7 to 1.2 ± 0.8 (P = 0.022) over 12 months.

Compared to controls, the TOF plus IGU group exhibited superior outcomes: baseline hemoglobin levels were significantly higher (112.3 vs. 97.9 g/L, *P* = 0.013), a difference that persisted at 12 months (117.0 vs. 121.4 g/L, *P* = 0.003). CRP reduction was more pronounced in the TOF plus IGU group at 3 months (8.5 vs. 20.2 mg/L, *P* = 0.002), and complete remission rates of RA improved faster (*P* < 0.001 at 3 months). However, between-group comparisons did not show statistically significant differences in clinical remission rates at 3, 6, or 12 months (**Table 3**). The TOF plus IGU cohort also demonstrated greater, DLCO SB% improvement at 3 months (61.1 ± 14.5% vs. 73.1 ± 19.6%, *P* = 0.045), lower HRCT fibrosis scores at 12 months (5.1 vs. 7.5, *P* = 0.004), and higher rates of imaging stability/regression (92.3% vs. 60.0%, *P* = 0.047). While TOF plus IGU led to a more rapid reduction in CRP at 3 months, both groups achieved comparable low-grade inflammation by 12 months (*P* = 0.217). Hemoglobin and platelet levels remained stable in both groups (*P* = 0.365 and *P* > 0.05, respectively), with no severe adverse events reported in the TOF plus IGU group (**Tables 3**, **4**).

**Table 3:** Longitudinal comparison of clinical indices, pulmonary function, and HRCT scores in patients with PF RA-ILD (n=28).

Variable	Group	Baseline 0	3 months	p (0-3)	6 months	p (0-6)	12 months	p (0-12)	P (Baseline)	P (3m)	P (6m)	P (12m)
RBC (×10¹²/L)	TOF+IGU	3.9 ± 0.5	3.7 ± 0.4	0.542	3.9 ± 1.6	0.271	4.1 ± 1.1	0.392	0.207	0.616	0.857	0.884
	b/csDMARDs	3.1 ± 2.1	3.9 ± 1.4		3.8 ± 1.6		4.0 ± 2.3					
HGB (g/L)	TOF+IGU	112 ± 14	116 ± 9	0.580	119 ± 4	0.246	117 ± 3	0.365	**0.013**	0.463	0.144	**0.003**
	b/csDMARDs	97.9 ± 16	118 ± 12		115 ± 10		121 ± 4					
WBC (×10^9^/L)	TOF+IGU	6.6 ± 1.5	8.1 ± 2.5	**0.038**	9.1 ± 1.9	**0.021**	9.0 ± 2.4	**0.020**	0.504	0.197	0.457	0.868
	b/csDMARDs	5.7 ± 5.2	6.6 ± 3.6		9.8 ± 3.2		9.2 ± 4.3					
PLT (×10^9^/L)	TOF+IGU	245 ± 48	225 ± 47	0.357	214 ± 37	0.479	224 ± 26	0.363	0.522	0.674	**0.055**	0.234
	b/csDMARDs	254 ± 40	232 ± 51		243 ± 43		237 ± 34					
RF (IU/mL)	TOF+IGU	79.7 ± 64	36.8 ± 35	**0.044**	29.8 ± 18	**0.031**	23.4 ± 22	**0.023**	0.564	0.739	0.837	0.581
	b/csDMARDs	68.9 ± 45	41.3 ± 40		31.2 ± 22		27.9 ± 21					
Anti-CCP (RU/mL)	TOF+IGU	157 ± 58	95.9 ± 59	**0.046**	110 ± 33	**0.028**	79.8 ± 33	0.231	0.314	0.822	0.073	0.737
	b/csDMARDs	138 ± 53	101 ± 64		89.7 ± 28		84.6 ± 44					
CRP (mg/L)	TOF+IGU	30.5 ± 23	8.5 ± 6.6	**0.024**	8.9 ± 11	**0.045**	5.1 ± 3.3	**0.033**	0.467	**0.002**	0.716	0.217
	b/csDMARDs	37.1 ± 26	20.2 ± 12		10.2 ± 9.6		6.9 ± 4.5					
ESR (mm/h)	TOF+IGU	46.2 ± 19	36.5 ± 15	**0.050**	26.3 ± 19	**0.047**	20.1 ± 19	**0.012**	0.077	0.133	0.202	0.183
	b/csDMARDs	58.9 ± 21	46.8 ± 22		33.6 ± 13		28.7 ± 21					
DAS28-CRP (CR), n(%)	TOF+IGU	0 (0)	3 (23.1)	**0.021**	5 (38.5)	**0.008**	7 (53.8)	**0.002**	–	0.914	0.723	0.887
	b/csDMARDs	0 (0)	4 (26.7)		6 (40.0)		8 (53.3)					
FVC (%)	TOF+IGU	79.5 ± 13	83.7 ± 16	0.079	85.3 ± 14	**0.008**	84.4 ± 12	**0.009**	0.656	0.722	0.217	0.212
	b/csDMARDs	77.3 ± 16	81.3 ± 20		79.2 ± 12		78.1 ± 15					
DLCO SB (%)	TOF+IGU	62.3 ± 12	61.1 ± 15	0.370	71.2 ± 11	**0.032**	70.5 ± 12	**0.04**	0.292	**0.045**	0.441	0.566
	b/csDMARDs	68.9 ± 21	73.1 ± 20		75.2 ± 16		73.5 ± 15					
HRCT: GGO score	TOF+IGU	4.9 ± 2.7	2.6 ± 0.6	**0.043**	1.3 ± 0.7	**0.025**	1.2 ± 0.8	**0.022**	0.595	0.541	0.385	0.248
	b/csDMARDs	5.5 ± 3.2	2.8 ± 1.1		1.7 ± 1.5		1.6 ± 1.0					
HRCT: Fibrosis score	TOF+IGU	9.6 ± 2.5	8.8 ± 3.1	0.640	6.2 ± 1.7	**0.044**	5.1 ± 1.6	**0.026**	0.282	0.624	0.101	**0.004**
	b/csDMARDs	10.9 ± 3.7	9.5 ± 4.5		8.6 ± 5.2		7.5 ± 2.3					
HRCT: D	TOF+IGU	13(100.0)	5(38.5)	**<0.001**	1 (7.7)	**<0.001**	1 (7.7)	**<0.001**	–	0.638	**0.042**	0.212
	b/csDMARDs	15(100.0)	8(53.3)		6(40.0)		5(33.3)					
HRCT: S+R, n(%)	TOF+IGU	0 (0)	8 (61.5)	**<0.001**	12 (92.3)	**<0.001**	12 (92.3)	**<0.001**	–	0.887	**0.047**	0.168
	b/csDMARDs	0 (0)	9 (60.0)		9 (60.0)		10 (66.7)					

Data are presented as mean ± standard deviation or n (%). Significant P-values (< 0.05) are in bold. p: the value for within-group comparison (each time point vs. baseline). P: the value for between-group comparison at the specified time point.

TOF, tofacitinib; IGU, iguratimod; b/csDMARDs, biologic/conventional synthetic disease-modifying antirheumatic drugs; RBC, red blood cells; HGB, haemoglobin; WBC, white blood cells; PLT, platelets; RF, rheumatoid factor; anti-CCP, anti-cyclic citrullinated peptide; CRP, C-reactive protein; ESR, erythrocyte sedimentation rate; DAS28-CRP, Disease Activity Score in 28 joints using C-reactive protein; CR, Complete Remission; FVC, forced vital capacity; DLCO SB, diffusing capacity for carbon monoxide; HRCT, high-resolution computed tomography; GGO, ground-glass opacity; D, deterioration; S, stability; R, regression.

**Table 4:** Longitudinal changes in clinical indices, pulmonary function, and HRCT scores.

Variable	Group	Baseline	3 Months	6 Months	12 Months	P (0-12 months)
Inflammatory markers						
CRP, mg/L	TOF+IGU	30.5 ± 23.1	8.5 ± 6.6**^†**	8.9 ± 10.9	5.1 ± 3.3	**0.033**
	Control	37.1 ± 26.4	20.2 ± 11.7	10.2 ± 9.6	6.9 ± 4.5	0.217
ESR, mm/h	TOF+IGU	46.2 ± 18.8	36.5 ± 14.6	26.3 ± 18.9	20.1 ± 18.9	**0.012**
	Control	58.9 ± 21.3	46.8 ± 22.3	33.6 ± 12.9	28.7 ± 21.1	0.183
Pulmonary function						
FVC, % predicted	TOF+IGU	79.5 ± 12.9	83.7 ± 15.5	85.3 ± 13.6**^†**	84.4 ± 11.5	**0.009**
	Control	77.3 ± 15.8	81.3 ± 19.8	79.2 ± 11.6	78.1 ± 14.6	0.212
DLCO SB, % predicted	TOF+IGU	62.3 ± 11.6	61.1 ± 14.5	71.2 ± 11.3	70.5 ± 12.1	**0.040**
	Control	68.9 ± 21.1	73.1 ± 19.6	75.2 ± 15.6	73.5 ± 15.2	0.566
HRCT scores						
Total GGO score	TOF+IGU	4.9 ± 2.7	2.6 ± 0.6	1.3 ± 0.7	1.2 ± 0.8	**0.022**
	Control	5.5 ± 3.2	2.8 ± 1.1	1.7 ± 1.5	1.6 ± 1.0	0.248
Total fibrosis score	TOF+IGU	9.6 ± 2.5	8.8 ± 3.1	6.2 ± 1.7	5.1 ± 1.6**^†**	**0.026**
	Control	10.9 ± 3.7	9.5 ± 4.5	8.6 ± 5.2	7.5 ± 2.3	**0.004**
Radiographic outcome, n (%)						
Stability+Regression	TOF+IGU	0 (0)	8 (61.5)	12 (92.3)	12 (92.3)**^†**	**<0.001**
	Control	0 (0)	9 (60.0)	9 (60.0)	10 (66.7)	0.168

Data are presented as mean ± standard deviation or n (%). P (0-12 months): within-group comparison from baseline to 12 months. Significant P-values (< 0.05) are in bold.

^†Significant between-group differences at the specified time point: CRP at 3 months (P = 0.002), FVC at 6 months (P = 0.008), fibrosis score at 12 months (P = 0.004), stability/regression at 12 months (P = 0.047).

CRP, C-reactive protein; ESR, erythrocyte sedimentation rate; FVC, forced vital capacity; DLCO SB, diffusing capacity for carbon monoxide single-breath method; HRCT, high-resolution computed tomography; GGO, ground-glass opacity.

In exploratory subgroup analysis, both UIP and NSIP patterns showed trends toward improvement in fibrosis scores, with no clear differential response, though small numbers preclude definitive conclusions.

## Discussion and conclusion

PF-RA ILD represents a therapeutic challenge due to its dual burden of articular and pulmonary pathology, compounded by the limited efficacy and potential toxicity of conventional therapies. While current guidelines conditionally recommend agents such as mycophenolate, rituximab, and cyclophosphamide (2023 ACR Guidelines) for systemic autoimmune rheumatic disease-associated ILD (SARD-ILD), real-world experience suggests variable articular response, and some conventional DMARDs prioritized for joint disease may lack efficacy or even pose risks in ILD (26, 27). Conversely, drugs effective for RA articular disease, such as methotrexate and TNF inhibitors, lack robust evidence for ILD management and may even exacerbate pulmonary fibrosis (27). This therapeutic dichotomy underscores the unmet need for agents capable of simultaneously targeting synovitis and lung fibrosis-a gap addressed by our pilot study evaluating the novel combination of TOF and IGU.

Our study demonstrates that TOF plus IGU achieved rapid and sustained systemic inflammation control, evidenced by reduction in CRP within 3 months (*P* = 0.002 versus controls). While this reduction occurred more rapidly than with conventional therapy, both groups attained comparable low-grade inflammation by 12 months, indicating that sustained inflammatory control is achievable with either strategy. Clinically, the TOF plus IGU regimen was associated with a progressive increase in CR rates, reaching 53.8% at 12 months. However, between-group comparisons showed no statistically significant differences in remission rates at most time points, suggesting that both approaches can facilitate effective joint disease control over time. Importantly, significant improvements in pulmonary outcomes were observed. In a cohort with baseline moderate-to-severe fibrosis (mean total score ~9–11), TOF plus IGU treatment led to a 7.3% increase in FVC% at 6 months (*P* = 0.008). Concurrently, HRCT scans showed marked reductions in fibrosis scores (from 9.6 ± 2.5 to 5.1 ± 1.6, *P* = 0.026) and ground-glass opacity scores (from 4.9 ± 2.7 to 1.2 ± 0.8, *P* = 0.022), reflecting structural and functional amelioration.

These dual articular-pulmonary benefits align with the distinct yet complementary mechanisms of TOF and IGU. As a JAK1/3 inhibitor, TOF disrupts the JAK/STAT pathway-a key driver of fibroblast activation and extracellular matrix deposition in pulmonary fibrosis-while concurrently suppressing synovial inflammation through IL-6 and interferon-γ inhibition (8, 9, 28). Mechanistically, JAK inhibitors like tofacitinib can repolarize macrophages from a pro-fibrotic (M2) towards a more balanced state, addressing both inflammation and fibrosis (10). Recently, some retrospective studies showed that JAK inhibitors are effective in slowing down fibrosis in RA-ILD and patients treated with TOF have the lowest incidence of RA-ILD compared with that treated with adalimumab, rituximab, abatacept, and tocilizumab (11, 29). This is corroborated by real-world evidence showing tofacitinib can stabilize pulmonary function (FVC%) in RA-ILD patients over a median follow-up of 12 months (16). These results were confirmed in another group of RA-ILD patients, including a UIP pattern treated with JAK inhibitors (15).

IGU amplifies this effect by modulating B-cell activity, reducing immunoglobulin production, and inhibiting pro-fibrotic mediators such as matrix metalloproteinase-9 (18, 19). The efficacy of IGU in improving lung function (FVC%, DLCO%) in RA-ILD when added to background therapy has been previously reported (20). The synergy of these mechanisms likely underlies the observed “dual treat-to-target” efficacy, wherein 92.3% of patients achieved radiographic stability/regression compared to 60.0% in the control group (*P* = 0.047).

The clinical relevance of these findings is further highlighted by the steroid-sparing potential of TOF plus IGU: among the TOF pus IGU patients using glucocorticoids at baseline, 46.2% (6/13) successfully discontinued steroid therapy during the study, with no severe infections or thromboembolic events reported. Regarding safety, the literature aligns with our observations. The combination therapy demonstrated good tolerability; aside from occasional herpes zoster, no patients discontinued treatment due to adverse effects, and all were able to reduce their steroid dose (30). Similarly, a large cohort study confirmed that JAK inhibitors maintain an expected safety profile in higher-risk RA-ILD patients, with no new safety signals observed. Respiratory tract infections were the most frequently reported adverse event (15). This safety profile contrasts favorably with anti-fibrotic agents like nintedanib and pirfenidone, which are limited by hepatotoxicity, photosensitivity, and lack of articular efficacy. Importantly, our imaging data provide mechanistic insights: the resolution of fine reticulation and GGO-markers of acute inflammation-preceded improvements in coarse reticulation, a hallmark of established fibrosis. The observed sequence of improvement-first in GGO and fine reticulation, later in coarse reticulation-supports the hypothesis that early anti-inflammatory intervention may attenuate fibrotic progression. However, radiographic relapse after treatment cessation likely reflects renewed inflammatory activity rather than irreversible fibrosis, underscoring the need for sustained therapy and caution in interpreting these patterns as definitive proof of fibrotic arrest.

Despite these promising results, our study has limitations. The limitations include its retrospective nature, small sample size (n=28), and short follow-up, preventing long-term or safety conclusions. The use of combination therapy (TOF plus IGU) and the lack of monotherapy arms mean the individual drug contributions and any synergism cannot be determined. Without monotherapy arms, we cannot delineate individual drug contributions or synergy. Published studies suggest both TOF and IGU have independent efficacy in RA and RA-ILD, respectively, but direct comparison with our combination regimen awaits controlled trials. Furthermore, The applicability to severe disease is unknown. Potential confounding exists, as over half of the control group received TNFi, a drug class potentially associated with ILD progression, which may have overestimated the combination’s benefit. These results necessitate confirmation from prospective, randomized trials.

In conclusion, this pilot study positions TOF plus IGU as a cost-effective, dual-target strategy for PF-RA ILD, addressing both synovitis and pulmonary fibrosis through complementary anti-inflammatory and anti-fibrotic mechanisms. In China, where biologic DMARDs remain prohibitively expensive, this combination offers a pragmatic alternative with favorable accessibility. Larger prospective studies are warranted to validate these findings, elucidate optimal dosing regimens, and explore synergies with anti-fibrotic agents in advanced disease.

## Data Availability

The original contributions presented in the study are included in the article/supplementary material. Further inquiries can be directed to the corresponding author/s.
